# Biomechanical Properties of Small-Size Hamstring Autografts

**DOI:** 10.7759/cureus.8728

**Published:** 2020-06-20

**Authors:** Edward Haupt, Kevin J OKeefe, Terry B Clay, Nicholas Kenney, Kevin W Farmer

**Affiliations:** 1 Orthopaedics and Rehabilitation, University of Florida, Gainesville, USA; 2 Orthopaedics, University of South Alabama, Mobile, USA; 3 Orthopaedics, Baptist Health Louisville, Louisville, USA

**Keywords:** acl repair, hamstring autografts, biomechanical properties

## Abstract

Purpose

To evaluate small-size hamstring (HS) autografts for biomechanical properties and determine a threshold diameter necessary for appropriate reconstruction.

Methods

In a controlled laboratory setting, biomechanical testing was performed upon 15 hamstring autografts. The grafts were divided into three groups by diameter, with five grafts each at diameter sizes of 6, 7, and 8 mm. Testing of the specimens was performed using an MTS 858 (Materials Testing System, Eden Prairie, MN). We determined load to failure by looking at the maximum load as well as the stiffness of the graft. Statistical analysis was performed via analysis of variance (ANOVA) testing with Tukey's post-hoc test and P-values set at 0.05.

Results

There was a significant difference in ultimate tensile strength for the different size grafts: 1990 +/- 302.42 N for 6 mm grafts (n=5), 2179 +/- 685.36 N for the 7 mm grafts (n=5), and 3074 +/- 781 N for 8 mm grafts (n=5). This was statistically significant for the group overall (p=0.039), as well as between the 6 mm and 8 mm grafts (p=0.044). Graft stiffness for the 6 mm grafts was 317 +/- 85 N (n=5), 288.6 +/- 66 for 7 mm grafts (n=5), and 428.053 +/- 83 for 8 mm grafts (n=5). This achieved statistical significance for the group overall (p =0.037) as well as between the 8 mm and 7 mm grafts.

Conclusions

The biomechanical data presented here demonstrate that graft diameter is highly correlated with ultimate tensile strength and stiffness.

Clinical relevance

When viewing this biomechanical data in conjunction with prior clinical data, consideration should be given for the supplementation of an HS autograft as the size decreases below 8 mm.

## Introduction

Anterior cruciate ligament (ACL) injuries are common, and surgical reconstruction of anterior cruciate ligament tears is the most frequent knee ligament reconstruction procedure performed in the United States [1]. Modern intra-articular reconstruction techniques have yielded excellent reconstructive results, however, subsequent graft tears and anterior cruciate ligament revision surgery remains a problem. Rates of revision surgery, regardless of graft used, are quoted from 5%-20%, however, the precise mode of failure and indication for revision has not been well-standardized in previous literature [2-4]. Recurrent or persistent instability is one of the leading causes of revision surgery. Young, active patients experience revision surgeries at increased rates, suggesting they are more likely to experience graft failure or are less tolerant of instability symptoms [5]. Magnusson et al. demonstrated in a clinical study that younger age and smaller graft diameter are both independently correlated with the increased rate of revision surgery, suggesting that a larger graft is less likely to need surgical revision [6].

A common reconstruction technique uses a hamstring tendon (HT) autograft for ACL graft reconstruction [7-14]. Historically, bone-patellar-tendon-bone (BPTB) grafts were thought to be biomechanically superior in resistance to tensile strain. However, HT autograft preparation techniques that increase the size of the graft have been shown to be equivocal or even superior to BPTB grafts in terms of strength [13-14]. The most frequent HT graft configuration is a quadruple strand graft consisting of a doubled gracilis tendon paired with a doubled semitendinosus graft. This quadruple strand HT graft has been shown to be biomechanically superior to BPTB grafts in tension when the mean graft diameter is 7.7 mm to 8.5 mm, which, notably, is similar in size to the BPTB graft [14-16].

Biomechanical cadaver studies have demonstrated that the mean biomechanical strength to resist the maximum tensile load of the native ACL is approximately 2160 N in young adults (age 22-35). As adults age, this maximum tensile strength has been shown to decrease. Stiffness of the native ACL, defined as the ultimate load at failure compared to the cross-sectional area of the ACL, also decreases with age [17-20].

Studies of HT autografts show the doubling of the gracilis or semitendinosus approach, with a tensile strength of 1550 and 2630 N, respectively, of the native ACL [19]. The quadruple bundle HT graft has a mean tensile strength of 4090 N, exceeding that of the native ACL. Doubling and quadrupling of hamstring grafts show obvious benefits to tensile strength. Additionally, significant heterogeneity exists in the patient population regarding hamstring autograft size, as well as harvesting and preparation techniques [18-21].

Although these prior studies demonstrate increasing biomechanical strength with increasing graft diameter, previous studies do not provide biomechanical evidence to support a specific ACL graft diameter for reconstruction. Thus, the purpose of this study is threefold: 1) to evaluate small size hamstring graft (6, 7, and 8 mm) for biomechanical qualities (ultimate tensile strength, stiffness, and creep), 2) to determine a threshold diameter of a looped graft necessary for appropriate reconstruction, and, thus, 3) to determine at what diameter should consideration be given to supplement an HS autograft.

## Materials and methods

There are no conflicts of interest to disclose from the corresponding authors of this manuscript. This project was funded internally by the University of Florida. This research involves cadaveric specimen testing utilizing tabletop biomechanical devices, and thus is exempt from Institutional Review Board approval.

In a controlled laboratory setting, biomechanical testing was performed upon 15 hamstring grafts. The hamstring grafts were harvested from cadaveric specimens of ages 50-70. Harvesting of the semitendinosus and gracilis tendons was performed using standard autograft techniques. A 3-cm longitudinal incision centered between the tibial tubercle and the posteromedial border of the tibia was utilized. The sartorius fascia was divided parallel to the superior border of the semitendinosus tendon and reflected inferomedially. The semitendinosus and gracilis tendons were identified on the deep surface and harvested with a tendon stripper. All grafts were stripped of muscle and were then pre-soaked in normal saline for 20 minutes. After preparation, the diameter of the graft was identified using standard sizing tubes in an ACL combo set. The grafts were pulled through the smallest-possible diameter tube without damage to the graft. The sizing procedure was confirmed and repeated three separate times.

The 15 hamstrings grafts were divided into three groups by diameter, with five grafts each at diameter sizes of 6, 7, and 8 mm. There were five quadruple strand grafts (four of which had diameter 8 mm, one had diameter 7 mm) and 10 double-strand grafts (five with 6 mm diameter, four with 7 mm diameter, and one with 8 mm diameter). Grafts were prepared to approximate similar tension across all strands.

**Figure 1:**
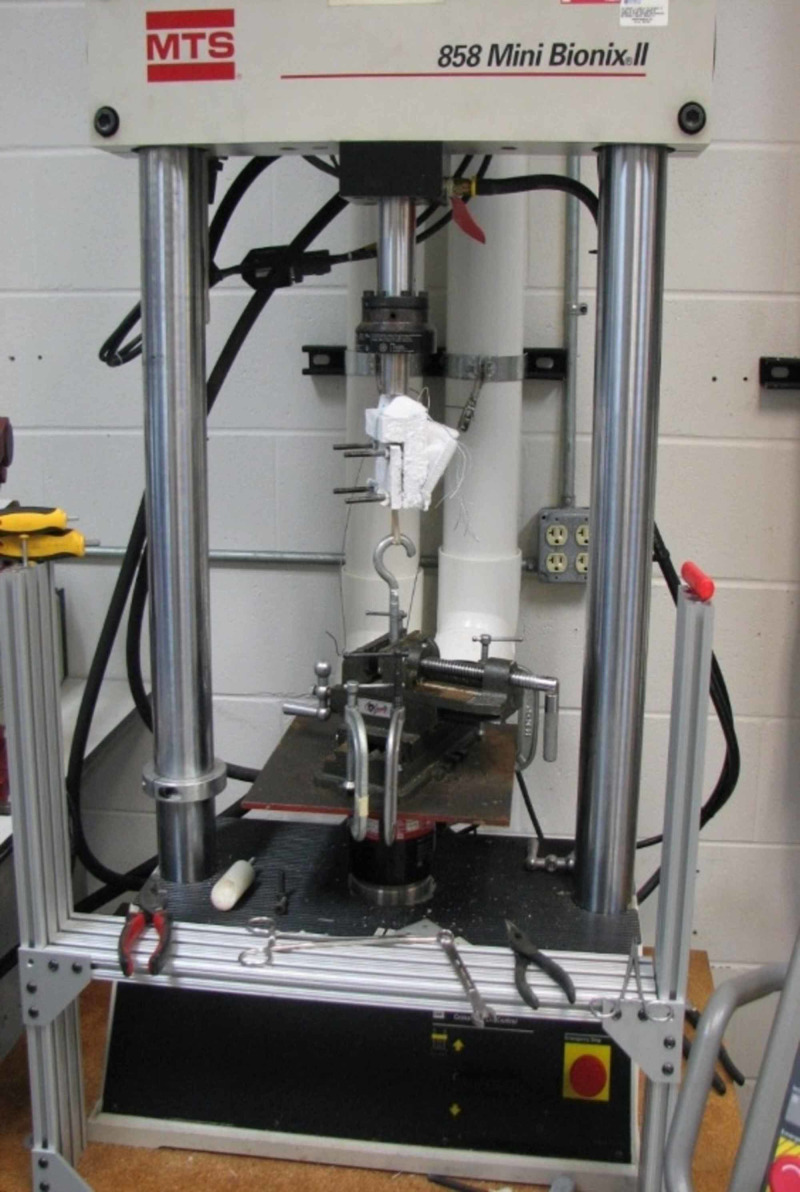
MTS 858 used for graft testing Testing of the specimens was performed using an MTS 858. The hamstring graft specimens were secured by the free end with clamps. They were then frozen into the clamps with dry ice before final tightening. Pictured is the block of dry ice holding the tendon secured to the hook. MTS 858: Materials Testing System, Eden Prairie, MN

**Figure 2:**
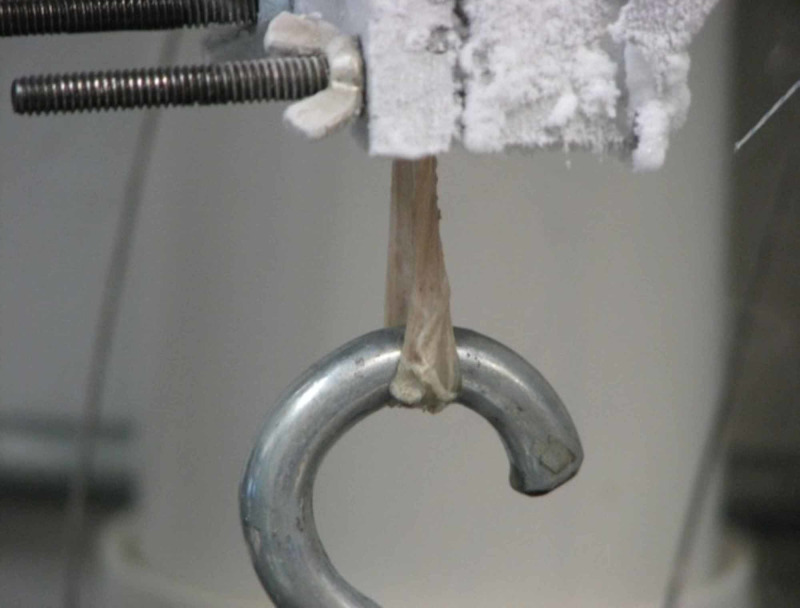
Close-up of testing device construct The hamstring graft specimens were secured by the free end with clamps. They were then frozen into the clamps with dry ice before final tightening. Pictured is a close-up of the block of dry ice holding the tendon secured to the hook.

Testing of the specimens was performed using a validated, commercially available materials testing device (MTS 858, Materials Testing System, Eden Prairie, MN). The hamstring graft specimens were secured by the free end with clamps. They were then frozen into the clamps with dry ice before final tightening (Figure 1). The looped end of the graft was secured with a hook where weight was applied (Figure 2).

In order to determine creep, we pre-tensioned the graft. We performed cyclic loading of 50-250 N @ 1 Hz for 1000 cycles. We determined load to failure by recording the peak load prior to graft rupture or subsequent load decrease, and stiffness of the graft was calculated by comparing the maximum load at failure to the cross-sectional area of the graft. All failures were mid substance. Statistical analysis was performed via analysis of variance (ANOVA) testing with Tukey’s post-hoc test and P-value set at 0.05.

## Results

**Figure 3:**
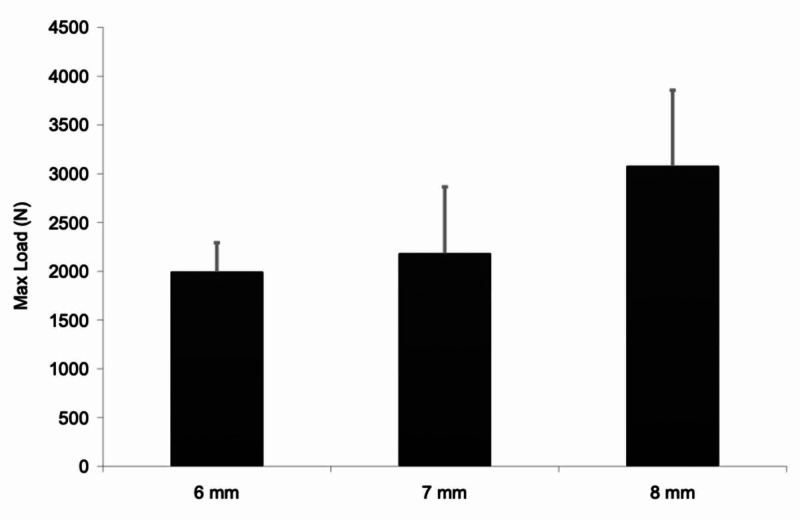
Maximum tensile load vs graft diameter Graph depicting the maximum tensile load identified versus the graft diameter

There was a significant difference in ultimate tensile strength (UTS) for the different size grafts: 1990+/-302.42N for 6 mm grafts (n=5), 2179 +/- 685.36N for 7 mm grafts (n=5), and 3074+/-781N for 8 mm grafts (n=5) (Figure 3, Table 1). Additionally, the difference between the 6 mm and 8 mm grafts was also found to be statistically significant (p=0.044) (Table 2).

**Table 1:** Average tensile load by diameter Table depicting the average tensile load (N) and standard deviation as determined by graft diameter in millimeters.

Max. force (N)	Size (mm)	Mean	Std. deviation	N
	6	1990.320	302.4223	5
	7	2179.440	685.3587	5
	8	3074.920	781.8207	5
	Total	2414.893	758.1160	15

**Table 2:** Comparison of average max force across 6, 7, 8 mm graft diameters Table depicting the comparison of graft diameter based upon mean tension. Statistical significance was noted between the smallest (6 mm) and largest (8 mm) grafts.

Dependent variable	(I) Size	(J) Size	Mean difference (I-J)	Std. error	Sig.
Max Force (N)	6	7	-189.120	395.3765	0.883
		8	-1084.600*	395.3765	0.044
	7	6	189.120	395.3765	0.883
		8	-895.480	395.3765	0.100
	8	6	1084.600*	395.3765	0.044
		7	895.480	395.3765	0.100

**Figure 4:**
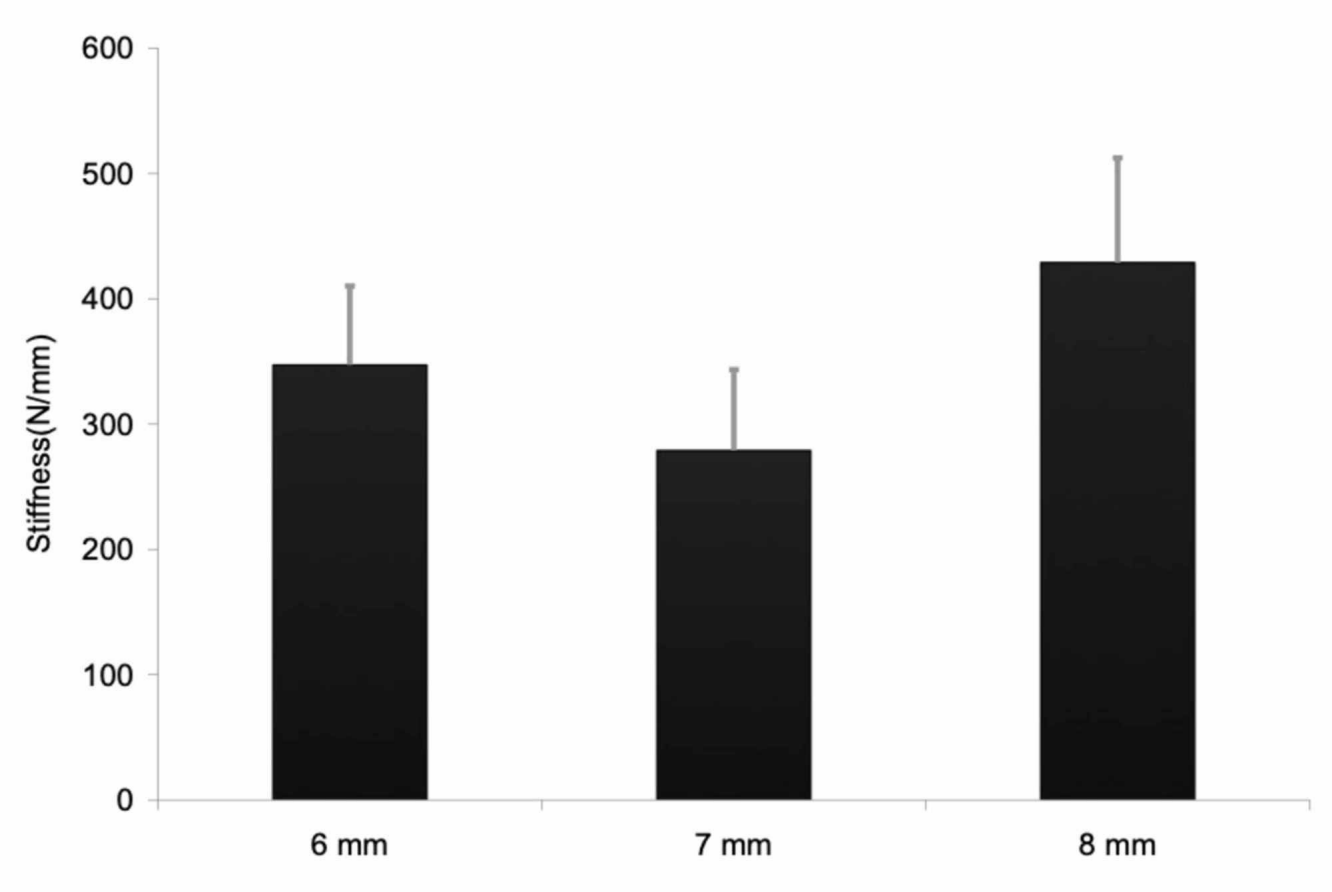
Graft stiffness by diameter Graph depicting graft stiffness by diameter

Graft stiffness for 6 mm grafts was 317+/-85N (n=5), 288.6+/-66 for 7 mm grafts (n=5), and 428.053+/-83 for 8 mm grafts (n=5) (Figure 4). This achieved statistical significance for the group overall (p=0.037) (Table 3) as well as between the 8 mm and 7 mm grafts (p=0.039) (Table 4).

**Table 3:** Graft stiffness statistical significance Table comparing the statistical significance of graft stiffness measurements.

Dependent variable		Sum of squares	df	Mean square	F	Sig.
Stiffness (N/mm)	Contrast	54704.841	2	27352.421	4.376	0.037
	Error	74999.076	12	6249.923		

**Table 4:** Comparison of graft stiffness across 6, 7, and 8 mm diameters Table comparing graft stiffness among different size grafts with statistical significance observed between the 7/8 mm grafts

Dependent variable	(I) Size	(J) Size	Mean difference (I-J)	Std. error	Sig.
Stiffness (N/mm)	6	7	29.180	49.9997	0.831
		8	-111.000	49.9997	0.108
	7	6	-29.180	49.9997	0.831
		8	-140.180*	49.9997	0.039
	8	6	111.000	49.9997	0.108
		7	140.180*	49.9997	0.039

Graft creep was measured as the difference in length at max force (250 N) between cycle 10 and cycle 1000. This did not reach statistical significance as a group or when comparing the 6, 7, and 8 mm grafts (Tables 5-7).

**Table 5:** Measurement of tendon graft creep Table showing the average change in graft length at max load after 1000 cycles of variable loading

Graft creep	Size (mm)	Mean	Std. deviation	N
	6	0.66406740	0.282114554	5
	7	0.81433100	0.323660640	5
	8	0.56054680	0.301467034	5
	Total	0.67964840	0.300445577	15

**Table 6:** Statistical analysis of measurements of tendon graft creep Table displays statistical analysis of measurements used to assess creep. Statistical difference was not reached as a group.

Dependent variable		Sum of squares	df	Mean square	F	Sig.
Graft Creep	Contrast	0.163	2	0.081	0.887	0.437
	Error	1.101	12	0.092		

**Table 7:** Comparison of tendon graft creep between 6, 7, and 8 mm graft diameters Table displaying a comparison of the measurements of tendon graft creep between different diameters. Statistical significance was not found between any graft diameter or as a group.

Dependent variable	(I) Size	(J) Size	Mean difference (I-J)	Std. error	Sig.
Graft Creep	6	7	-0.15026360	0.191564508	0.719
		8	0.10352060	0.191564508	0.853
	7	6	0.15026360	0.191564508	0.719
		8	0.25378420	0.191564508	0.409
	8	6	-0.10352060	0.191564508	0.853
		7	-0.25378420	0.191564508	0.409

## Discussion

Previous data demonstrated that quadruple-strand grafts have superior strength versus maximum loads when compared to double or single strand grafts, and graft cross-sectional area has been strongly correlated with graft strength [18]. Studies comparing BPTB to HT grafts have also identified that HT grafts are equivalent in tensile strength to patellar tendon grafts if the diameter of the HT graft is of similar size [13].

Our study demonstrated that the UTS of HT grafts of diameter 8 mm was greater than or equal to that of the native ACL. In addition, the difference in UTS between 6 and 8 mm grafts was statistically significant. The difference in 6 and 7 mm grafts did not reach statistical significance although there was a trend toward suboptimal strength (Table 8). In regards to stiffness, the mean for each size was greater than the native ACL, 242±28 N/mm, as determined by Woo in his biomechanical study [19]. This could possibly be attributed to the age of the grafts as well as an effect of the cooling process.

**Table 8:** Summary of max force and stiffness across 6, 7, and 8 mm diameters Summary of the data including max force and stiffness across the graft diameters of 6, 7, and 8 mm with included 95% confidence intervals.

Dependent variable				95% confidence interval	
	Size (mm)	Mean	Std. Error	Lower Bound	Upper Bound
Max Force (N)	6	1990.320	279.573	1381.182	2599.458
	7	2179.440	279.573	1570.302	2788.578
	8	3074.920	279.573	2465.782	3684.058
Stiffness (N/mm)	6	317.780	35.355	240.748	394.812
	7	288.600	35.355	211.568	365.632
	8	428.780	35.355	351.748	505.812

The biomechanical data of our study would support the clinical data published by Magnusson et al., which demonstrated that smaller diameter HT grafts had increased rates of revision [6]. Grafts >8 mm had a 1.7% revision rate (n=1/58) versus 6.5% (n=9/139) in patients with grafts between 7 mm and 8 mm, and 13.6% (8 of 59) with grafts 7 mm or less in diameter. The population in the study by Magnusson notably used quadruple-strand grafts only in comparison to our study, which used both double and quadruple strand grafts. Previous literature has identified that grafts should be at least 7 mm in diameter, and our study confirms that a 7 mm graft approaches the properties of the native ACL. However, the biomechanical data presented here confirm that an 8 mm graft is stronger. This viewed in conjunction with the prior clinical data by Magnusson et al., which indicated higher revision rates for grafts smaller than 8 mm, would argue for an optimal graft diameter of at least 8 mm.

Some prior studies have also attributed gender as an independent risk factor for revision when undergoing HT autografts [22-25]. Ma et al. demonstrated that female patients undergoing an autograft HT harvest are nearly twice as likely to have grafts smaller than 8 mm in diameter in comparison with male patients (40% of females compared to 19% of males) [26]. Magnusson et al. identified that there was no difference in the ACL revision rate between male and female patients when graft diameter was equivalent [6]. Many of the studies which have reported a higher rate of revision among female patients either do not report HT graft diameter or do not control it. Thus, this information in combination with our biomechanical data could attribute smaller HT graft size as the contributing factor for the increased rate of ACL revision surgery for women [3,23-25].

The limitations of our study include our sample size of harvested tendon-grafts, the cadaver age for harvested tendons and resulting tendon variability, and the method of testing tensile strength. Additionally, prior studies demonstrate that the weakest part of a reconstruction construct are the bony fixation points [27-28]. Our graft failures were mid substance and thus should be taken into consideration when viewing ultimate tensile strength and stiffness.

## Conclusions

ACL reconstruction surgery is one of the most common ligamentous reconstruction procedures. Autologous hamstring grafting is an attractive choice for reconstruction. Previous clinical data has shown smaller grafts have an increased rate of revision surgery. The biomechanical data presented here demonstrate that graft diameter is highly correlated with ultimate tensile strength and stiffness. When viewing this biomechanical data in conjunction with prior clinical data, consideration should be given for the supplementation of HS autografts that fall below 8 mm.
